# Optimization of a TEEDA–based initiation system for SDS–PAGE

**DOI:** 10.3389/abp.2026.17215

**Published:** 2026-08-12

**Authors:** Chaoyang Chen, Mengting Sun, Xiaoxia Ji, Lin Ge, Jia Xu, Mengyuan Cai

**Affiliations:** 1 Department of Medical Science and Technology, Suzhou Chien–Shiung Institute of Technology, Taicang, China; 2 Jiangsu Province Engineering Research Center of Novel Tumor–targeting Drug Conjugates, Taicang, China; 3 School of Pharmacy & School of Biological and Food Engineering, Changzhou University, Changzhou, China; 4 Crown Bioscience Inc., Taicang, China

**Keywords:** gel polymerization, polyacrylamide gel, SDS-PAGE, sodium bisulfite, TEEDA

## Abstract

Polyacrylamide gel preparation for SDS–PAGE is conventionally initiated by the ammonium persulfate (APS)–TEMED system. Although effective, the relatively high volatility of TEMED may increase operator exposure during routine laboratory use. In this study, N,N,N′,N–tetraethylethylenediamine (TEEDA) was systematically evaluated as an alternative accelerator for SDS–PAGE gel preparation. TEEDA alone produced polymerization and protein separation performance comparable to those of the conventional TEMED–APS system, although higher concentrations (8.3 mM in 10% separating gels and 17.4 mM in 5% stacking gels) were required than those of TEMED (4.0 mM and 6.7 mM, respectively). When TEEDA concentrations comparable to those of TEMED were used, supplementation with 0.1% SBS in separating gels and 0.01% SBS in stacking gels restored polymerization and protein separation performance to levels comparable with those of the conventional TEMED–APS system. These findings provide an optimized TEEDA–based formulation for routine SDS–PAGE.

## Introduction

Sodium dodecyl sulfate–polyacrylamide gel electrophoresis (SDS–PAGE) is a widely used analytical technique in protein biochemistry for protein separation, molecular weight estimation, purity assessment, and sample preparation prior to Western blotting (Brunelle and Green, 2014). Conventional SDS–PAGE gel polymerization is initiated by the N,N,N′,N′–tetramethylethylenediamine (TEMED)–ammonium persulfate (APS) system, which originated from the disc electrophoresis methodology (Davis, 1964) and was later adapted for SDS–PAGE (Laemmli, 1970), as implemented in standard protocols (Gallagher, 2007). TEMED accelerates the decomposition of APS to generate free radicals, thereby initiating acrylamide polymerization via a persulfate–amine electron–transfer process (Hunkeler, 1991). Owing to its efficiency and simplicity, the TEMED–APS system has remained the standard gel initiation method for decades. Beyond the standard Laemmli system, alternative discontinuous buffers such as the Tricine–SDS system have been developed to extend resolution to the 1–100 kDa range, particularly for proteins <30 kDa (Schägger and von Jagow, 1987; Schägger, 2006).

Despite its widespread use, TEMED has several practical disadvantages (Pumford et al., 2024). It is highly volatile, has a strong irritating odor during gel preparation, and is classified as an acute toxicity Category 3 substance for oral and inhalation exposure (National Center for Biotechnology Information, 2026a). These characteristics may reduce user comfort and increase the risk of repeated laboratory exposure.

N,N,N′,N′–tetraethylethylenediamine (TEEDA) is a structural analogue of TEMED in which the methyl substituents are replaced by ethyl groups. Compared with TEMED, TEEDA has a higher boiling point (>190 °C), and lower acute inhalation toxicity (National Center for Biotechnology Information, 2026b), making it a potentially safer amine accelerator for routine laboratory applications.

Although TEEDA is commercially available and has occasionally been mentioned as a potential substitute for TEMED, detailed formulations and optimization strategies for preparing SDS–PAGE gels with TEEDA have rarely been reported. Likewise, although several tertiary amines have historically been evaluated as substitutes for TEMED (Davis, 1964), detailed formulations suitable for routine SDS-PAGE gel preparation have rarely been reported. Therefore, optimization of practical TEEDA-based gel formulations remains necessary.

The present study systematically optimized the use of TEEDA as an alternative to TEMED for SDS–PAGE. The effects of TEEDA concentration and APS–sodium bisulfite (SBS)–assisted initiation on gel polymerization were evaluated, and the resulting gels were further assessed for protein separation performance. The objective of this study was to establish an optimized TEEDA–based gel preparation method for routine SDS–PAGE applications.

## Materials and methods

### Materials

The following reagents were obtained from the indicated sources: N,N,N′,N′–tetraethylethylenediamine (TEEDA; purity = 99.08%, Density = 0.805 g/mL, Shanghai Aladdin Biochemical Technology Co., Ltd.); sodium bisulfite (SBS; Analytical grade, Hunan Xianghong Food Additives Co., Ltd.); N,N,N′,N′–tetramethylethylenediamine (TEMED; Purity = 99.82%, Density = 0.775 g/mL, Beyotime Biotechnology Co., Ltd.); ammonium persulfate (APS; Analytical grade, Greagent). Tris–HCl buffers (pH 6.8 and 8.8), SDS–PAGE loading buffer, pre–stained protein markers (10–180 kDa), and 30% acrylamide–bisacrylamide solution (29:1) were obtained from Beyotime Biotechnology Co., Ltd. Ammonium sulfate, Methanol, acetic acid, glycine, SDS, Tris base, and Coomassie Brilliant Blue G–250 were purchased from Sinopharm Chemical Reagent Co., Ltd. Deionized water (ddH_2_O) was prepared in–house. Electrophoresis was performed using a Mini–PROTEAN Tetra Cell system (Bio–Rad). Vero cells were kindly provided by Dr. Zhiyong Li.

### Protein sample preparation

Egg white proteins were fractionated by ammonium sulfate precipitation. Briefly, egg white was diluted 10–fold with ddH_2_O, filtered through four layers of gauze, and mixed with 1.5 volumes of saturated ammonium sulfate solution. After centrifugation at 8,000 rpm for 10 min, the precipitate was collected and dissolved in ddH_2_O (Fraction 1). Solid ammonium sulfate was then added to the supernatant until marked turbidity appeared. After centrifugation at 8,000 rpm for 10 min, the precipitate was collected and dissolved in ddH_2_O to obtain Fraction 2. *Escherichia coli* cells from overnight culture were harvested by centrifugation and resuspended in SDS–PAGE loading buffer. Vero cells were washed with phosphate–buffered saline and lysed directly in SDS–PAGE loading buffer. All samples were mixed with 5× loading buffer (4:1, v/v, sample/buffer), heated at 100 °C for 10 min, and cooled to room temperature prior to electrophoresis. *Escherichia coli* and Vero cell lysates were briefly centrifuged (12000 rpm, 1 min) to remove insoluble debris before electrophoresis.

### SDS–PAGE gel preparation and polymerization time determination

Conventional SDS–PAGE gels initiated by the TEMED–APS system served as controls throughout this study (Supplementary Table S1). The formulation was adapted from standard protocols (Laemmli, 1970; Gallagher, 2007). Separating and stacking gels contained 10% and 5% acrylamide, respectively, with final volumes of approximately 5 mL and 4 mL. The final TEMED concentrations were 4.0 mM in separating gels and 6.7 mM in stacking gels.

To evaluate of TEEDA as a substitute for TEMED, TEEDA concentrations ranging from 1.2 to 8.3 mM were tested in separating gels and from 6.8 to 17.4 mM in stacking gels, while all other components were kept constant (Supplementary Table S2). For evaluation of the TEEDA–SBS–APS initiation system, TEEDA concentrations were fixed at approximately 4.1 mM in separating gels and 6.8 mM in stacking gels, whereas SBS concentrations ranged from 0% to 7% (w/v) in separating gels and from 0% to 1% (w/v) in stacking gels (Supplementary Table S3). The total volume was kept constant by adjusting ddH_2_O.

Polymerization was initiated by the addition of freshly prepared APS. Fresh APS and SBS solutions were prepared immediately before use and used within 2 h. Gel mixtures were prepared in 15 mL polypropylene centrifuge tubes and allowed to polymerize at 20 °C–27 °C under a relative humidity of 40%–45%. Polymerization time was defined as the interval from APS addition to the point at which the gel solution no longer flowed upon gentle tilting of the centrifuge tube rack. All measurements were performed by the same operator under identical experimental conditions using this predefined endpoint. During gel preparation, the polymerized gels were routinely inspected for visible defects, including bubbles, brittle gels, uneven interfaces between the stacking and separating gels, and incomplete polymerization. None of these defects were observed under the conditions that produced successful gel polymerization.

### SDS–PAGE electrophoresis and staining

Protein samples (10 μL) were loaded onto the gels (1.0 mm spacer, 8.6 × 6.8 cm). Electrophoresis was performed at 80 V through the stacking gel and 160 V through the separating gel until the bromophenol blue dye front reached the bottom of the gel. Gels were stained with Coomassie Brilliant Blue G–250 and destained with methanol/acetic acid solution until clear protein bands were obtained. Gel images were acquired using a Gel Doc Go Imaging System (Bio–Rad, Hercules, CA, USA).

### Statistical analysis

All polymerization experiments were performed independently three times. Data are presented as mean ± standard deviation (SD). Statistical analyses were performed using GraphPad Prism 10.0 (GraphPad Software, San Diego, CA, USA). Differences among groups were analyzed by one–way analysis of variance (ANOVA) followed by Dunnett’s multiple–comparison test using the conventional TEMED–APS system as the control. Differences were considered statistically significant at *P* < 0.05.

## Results and discussion

### Effect of TEEDA concentration on polymerization of separating and stacking gels

**FIGURE 1:**
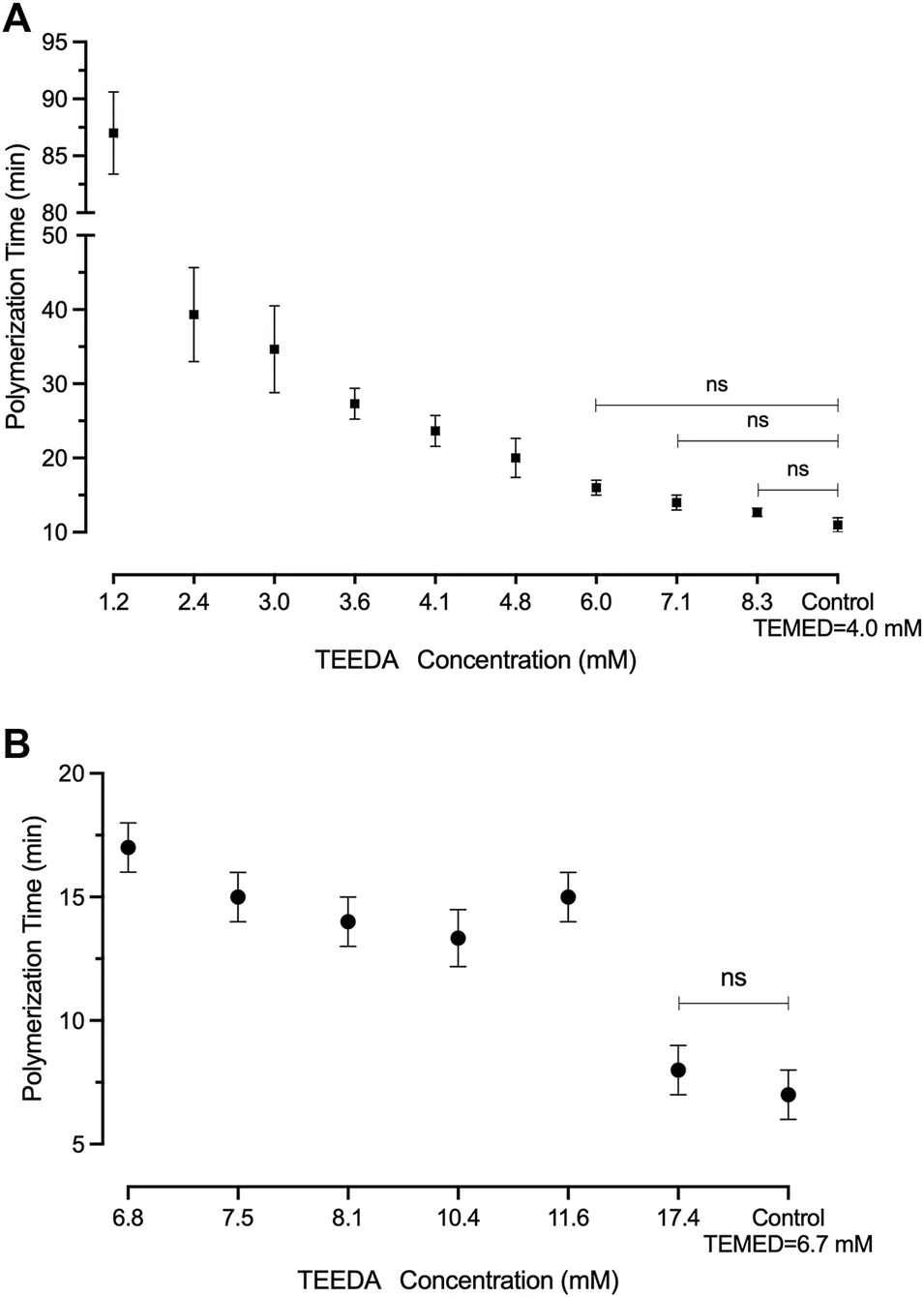
Effect of TEEDA concentration on SDS–PAGE gel polymerization. **(A)** Polymerization time of 10% separating gels (approximately 5 mL total volume) prepared with different concentrations of TEEDA. **(B)** Polymerization time of 5% stacking gels (approximately 4 mL total volume) prepared with different concentrations of TEEDA. Data are presented as mean ± SD (n = 3). Statistical analysis was performed using one–way ANOVA followed by Dunnett’s multiple–comparison test, with the conventional TEMED–APS system as the control. ns, *P* > 0.05; all other groups differed significantly from the control (*P* < 0.05).

TEEDA was first evaluated as a substitute for TEMED in APS–initiated SDS–PAGE gel. In separating gels, polymerization time decreased progressively with increasing TEEDA concentration (Figure 1A), indicating a concentration–dependent acceleration of gelation. One–way ANOVA showed a significant effect of TEEDA concentration on polymerization time (F (9, 20) = 143.7, *P* < 0.0001). Dunnett’s multiple–comparison test, using the conventional TEMED–APS system (4.0 mM TEMED) as the control, demonstrated that polymerization remained significantly slower at TEEDA concentrations below 6.0 mM (*P* < 0.05), whereas no significant difference was observed at 6.0–8.3 mM (*P* > 0.05). These results indicate that increasing the TEEDA concentration achieved polymerization kinetics comparable to those of the conventional TEMED–APS system.

A similar concentration–dependent trend was observed in stacking gels (Figure 1B). Polymerization time differed significantly among the tested TEEDA concentrations (one–way ANOVA, F (6, 14) = 40.9, *P* < 0.0001). Compared with the TEMED–APS control (6.7 mM TEMED), only the highest TEEDA concentration tested (17.4 mM) produced a comparable polymerization time, whereas all lower concentrations (6.8–11.6 mM) resulted in significantly slower gelation (*P* < 0.01).

Overall, polymerization time was inversely correlated with TEEDA concentration in both separating and stacking gels. However, higher TEEDA concentrations were required to achieve polymerization rates comparable to those of TEMED, suggesting that TEEDA is a less efficient accelerator when used alone. This observation is consistent with the early work of Davis (Davis, 1964), who noted that although several tertiary amines could initiate acrylamide polymerization, TEMED was selected because of its superior accelerating efficiency. A similar explanation has been proposed by Hunkeler, who described persulfate-initiated acrylamide polymerization as involving donor-acceptor interactions between persulfate and tertiary amines (Hunkeler, 1991). Structural differences between TEEDA and TEMED may influence these interactions and consequently alter the efficiency of free-radical generation, although the underlying mechanism remains to be investigated.

Interestingly, the optimal TEEDA concentration differed markedly between separating and stacking gels. This difference is likely related to differences in gel composition, including pH, buffer composition, and acrylamide concentration, all of which can influence free–radical generation and polymerization kinetics. Therefore, TEEDA concentration should be optimized separately for separating and stacking gels rather than directly extrapolated from one gel formulation to another.

Taken together, these results indicate that TEEDA can serve as a practical substitute for TEMED for SDS–PAGE gel, although higher concentrations are required to achieve polymerization behavior comparable to that of the conventional TEMED–APS system.

### Effect of SBS on polymerization of TEEDA–initiated separating and stacking gels

**FIGURE 2:**
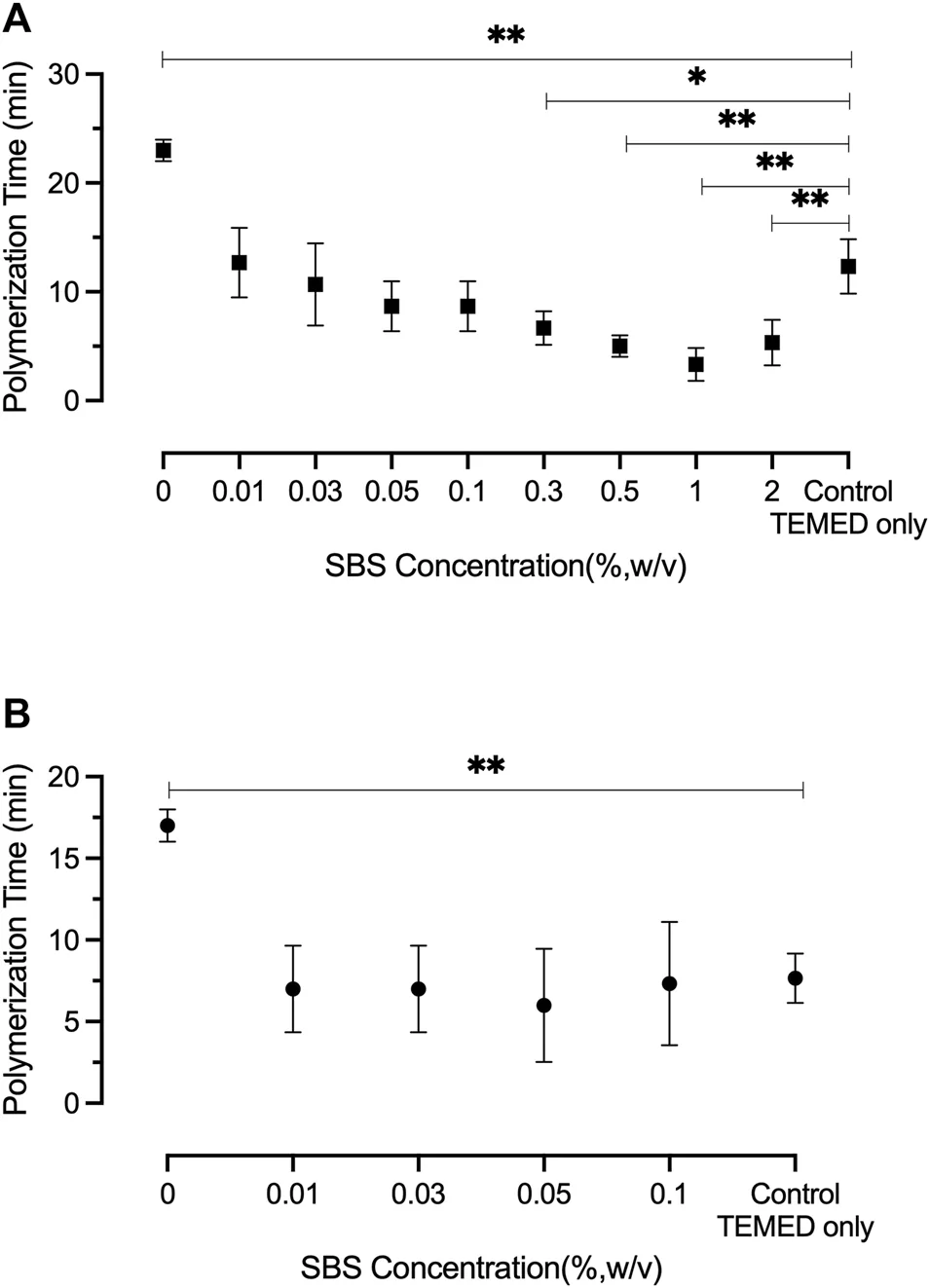
Effect of SBS concentration on the polymerization time of TEEDA–initiated SDS–PAGE gels. **(A)** Polymerization time of 10% separating gels (approximately 5 mL total volume) prepared with different concentrations of SBS. **(B)** Polymerization time of 5% stacking gels (approximately 4 mL total volume) prepared with different concentrations of SBS. Data are presented as mean ± SD (n = 3). Statistical analysis was performed using one–way ANOVA followed by Dunnett’s multiple–comparison test, with the conventional TEMED–APS system as the control. **, *P* < 0.01 versus the control; *, *P* < 0.05 versus the control; unmarked groups are not significantly different from the control (*P* > 0.05). Conditions in which no gelation occurred within 24 h are not shown.

To further improve the polymerization efficiency of the TEEDA–APS system, SBS was introduced while maintaining the TEEDA concentration at a level comparable to that of the conventional TEMED control. In separating gels, polymerization time decreased progressively with increasing SBS concentration from 0% to 1% (w/v), reaching the shortest gelation time at 1% SBS (Figure 2A). One–way ANOVA revealed a significant effect of SBS concentration on polymerization time (F (9, 20) = 18.14, P < 0.0001). Dunnett’s multiple–comparison test, showed that polymerization times at 0%, 0.3%, 0.5%, 1%, and 2% SBS differed significantly from the control (*P* < 0.05). At higher SBS concentrations (3% and 7%), the separating gel underwent incomplete polymerization or failed to polymerize within 24 h (data not shown).

A similar concentration–dependent effect was observed in stacking gels (Figure 2B). Polymerization time differed significantly among the tested SBS concentrations (one–way ANOVA, F (5, 12) = 6.998, *P* = 0.0028). Compared with the conventional TEMED–APS system, stacking gels containing 0.01%–0.1% SBS exhibited polymerization time that were not significantly different from the control (*P* > 0.05), whereas the gel without SBS polymerized significantly more slowly (*P* < 0.05). In contrast, no gelation was observed within 24 h at SBS concentrations of 0.3%, 0.5%, or 1% (data not shown).

These findings demonstrate that SBS exerts a concentration–dependent effect on TEEDA–initiated gel polymerization. The accelerating effect of SBS is consistent with the well-established persulfate-bisulfite redox initiation system, in which bisulfite promotes persulfate decomposition and increases the generation of initiating radicals (Sarac, 1999). Interestingly, excessive SBS inhibited gel formation in both separating and stacking gels. A possible explanation is that an excess of reducing agent alters the balance between radical generation and radical termination, thereby decreasing the concentration of effective propagating radicals required for acrylamide polymerization. In addition, the inhibition threshold differed markedly between separating and stacking gels, suggesting that the optimal SBS concentration is influenced by gel composition, including acrylamide concentration, buffer composition, and pH.

Collectively, these results indicate that appropriate SBS concentrations can substantially modify the polymerization behavior of TEEDA–initiated gels. However, whether the formulations exhibiting the shortest polymerization time also provide optimal electrophoretic performance requires further evaluation.

### Protein separation performance of gels prepared with the TEEDA–APS and TEEDA–SBS–APS systems

**FIGURE 3:**
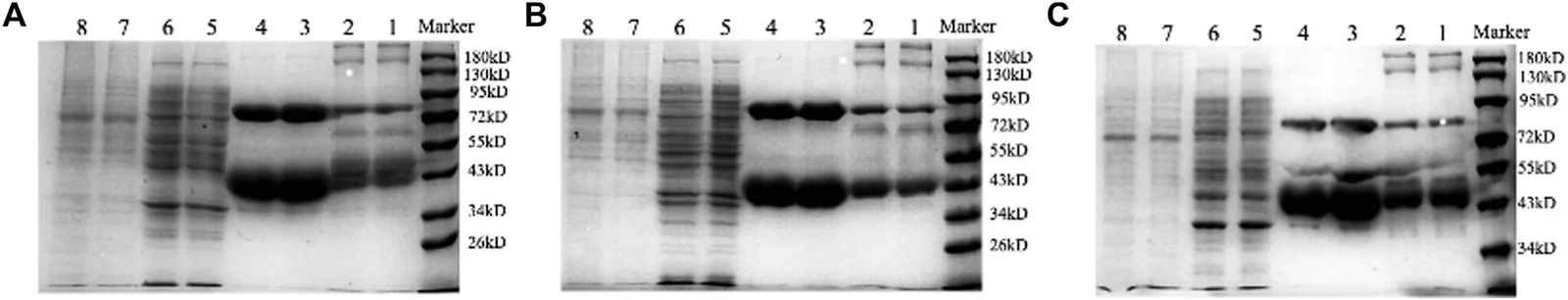
Protein separation performance of SDS–PAGE gels prepared using the TEMED–APS, TEEDA–APS, and TEEDA–SBS–APS initiation systems. Proteins were separated by SDS–PAGE and visualized by Coomassie Brilliant Blue staining. **(A)** Conventional TEMED–APS system (4.0 mM TEMED in the separating gel and 6.7 mM TEMED in the stacking gel). **(B)** TEEDA–APS system (8.3 mM TEEDA in the separating gel and 17.4 mM TEEDA in the stacking gel). **(C)** TEEDA–SBS–APS system (4.1 mM TEEDA plus 1% SBS in the separating gel and 6.8 mM TEEDA plus 0.01% SBS in the stacking gel). The acrylamide concentrations of the separating and stacking gels were 10% and 5%, respectively. Lanes 1–2, chicken egg white fraction 1; lanes 3–4, chicken egg white fraction 2; lanes 5–6, *Escherichia coli* lysates; lanes 7–8, Vero cell lysates. The pre-stained protein marker (Marker) contained bands at 17, 26, 34, 43, 55, 72, 95, 130, and 180 kDa. Representative gels from three independent experiments are shown.

The electrophoretic performance of the proposed initiation systems was evaluated using egg white proteins, *E. coli* lysates, and Vero cell lysates as model samples. Gels prepared with the optimized TEEDA–APS system (8.3 mM TEEDA in the separating gel and 17.4 mM TEEDA in the stacking gel) exhibited polymerization behavior comparable to that of the conventional TEMED–APS system. As shown in Figure 3B, protein bands were clear, well resolved, and comparable to those obtained using the conventional TEMED–APS system (Figure 3A), indicating that TEEDA can effectively replace TEMED without compromising electrophoretic performance when an appropriate concentration is used. Compared with commercial fast-casting gels, the present method does not eliminate manual gel preparation but provides a practical alternative formulation for laboratories that routinely cast their own gels and wish to avoid the use of TEMED.

The TEEDA–SBS–APS system was subsequently evaluated using the formulation that exhibited the shortest polymerization time in Section *Effect of SBS on polymerization of TEEDA–initiated separating and stacking gels* (1% SBS in the separating gel and 0.01% SBS in the stacking gel). Although this formulation produced satisfactory gel polymerization, its electrophoretic performance was inferior to that of the conventional system (Figure 3C). In particular, low–molecular–weight proteins were poorly resolved, fewer protein bands were detected, and the protein band at approximately 26 kDa was no longer detectable. In addition, protein migration through the separating gel was slower, resulting in an approximately 15 min longer electrophoresis time than the control.

In the discontinuous buffer system of SDS-PAGE, protein separation depends on the establishment of a stable ion boundary between chloride and glycine ions (Ornstein, 1964; Laemmli, 1970). Introducing 1% SBS into the separating gel substantially increases the concentration of sulfite species, which may alter the ionic composition and local buffering capacity of the gel. Such changes are expected to perturb the chloride-glycine ion boundary, resulting in reduced electrophoretic field uniformity. Consistent with this interpretation, low-molecular-weight proteins exhibited the greatest loss of resolution, and protein migration through the separating gel was delayed by approximately 15 min. Similar effects have been reported in other discontinuous electrophoretic systems in which modification of trailing ions alters stacking behavior and band resolution (Schägger and von Jagow, 1987).

**FIGURE 4:**
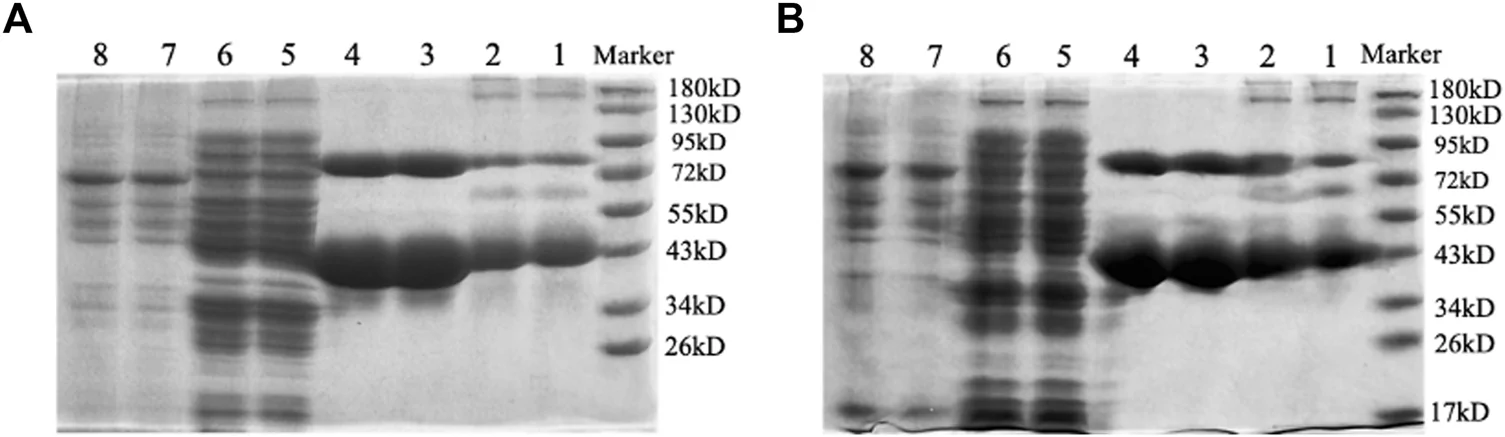
Protein separation performance of the optimized TEEDA–SBS–APS initiation system. Proteins were separated by SDS–PAGE and visualized by Coomassie Brilliant Blue staining. **(A)** Conventional TEMED–APS system (4.0 mM TEMED in the separating gel and 6.7 mM TEMED in the stacking gel). **(B)** Optimized TEEDA–SBS–APS system (4.1 mM TEEDA plus 0.1% SBS in the separating gel and 6.8 mM TEEDA plus 0.01% SBS in the stacking gel). The separating and stacking gels contained 10% and 5% acrylamide, respectively. Lanes 1–2, chicken egg white fraction 1; lanes 3–4, chicken egg white fraction 2; lanes 5–6, *Escherichia coli* lysates; lanes 7–8, Vero cell lysates. The pre-stained protein marker (Marker) contained bands at 17, 26, 34, 43, 55, 72, 95, 130, and 180 kDa. Representative gels from three independent experiments are shown.

To restore electrophoretic performance, the SBS concentration in the separating gel was reduced to 0.1% while maintaining 0.01% SBS in the stacking gel. Under these conditions, protein separation was restored to a level comparable to that of the conventional TEMED–APS system (Figures 4B versus Figure 4A), with no obvious differences in band resolution or migration pattern among the tested protein samples. At 0.1% SBS, the bisulfite pool is too small to depress the gel pH appreciably, thereby preserving the normal ion boundary required for efficient protein separation.

The low SBS concentration used in the stacking gel (0.01%) did not noticeably affect electrophoretic performance. This is consistent with the lower pH of the stacking gel, where glycine remains predominantly zwitterionic and the discontinuous buffer system is less susceptible to perturbation by trace amounts of bisulfite (Ornstein, 1964). These findings demonstrate that optimization of the TEEDA–SBS–APS system must consider electrophoretic performance in addition to gel polymerization kinetics.

Despite the encouraging results, several limitations should be acknowledged. First, TEEDA concentrations higher than those evaluated here were not investigated, and whether further increases improve polymerization or electrophoretic performance remains unknown. Second, the performance of the APS-SBS system in the absence of TEEDA was not examined, making it difficult to distinguish the individual contributions of TEEDA and SBS. Finally, only Coomassie Brilliant Blue staining was evaluated. The compatibility of the optimized formulations with downstream applications, particularly Western blotting, requires further investigation.

## Conclusion

TEEDA can serve as an effective alternative to TEMED for APS–initiated SDS–PAGE gel preparation when appropriate formulations are employed. By systematically optimizing the concentrations of TEEDA and SBS for separating and stacking gels, this study established TEEDA–based gel formulations that provide polymerization behavior and electrophoretic performance comparable to those of the conventional TEMED–APS system. These optimized formulations provide a practical reference for laboratories that routinely prepare SDS-PAGE gels in-house and are interested in adopting TEEDA as an alternative accelerator.

## Data Availability

The original contributions presented in the study are included in the article/Supplementary Material, further inquiries can be directed to the corresponding author.
