# Supplementary material for: Optimization of a TEEDA–based initiation system for SDS–PAGE
Source: Acta Biochim Pol. 2026 Aug 12;73:17215. doi: 10.3389/abp.2026.17215 (PMC13509136; doi:10.3389/abp.2026.17215)
Supplement: Supplementary file 1 [file Table1.docx]

**Table S1. Composition of SDS-PAGE gels prepared with APS and TEMED.**

|  | TEMED | | ddH_2_O | 30% ACR^*^ | Tris-HCl ^$^ | 10%SDS | 10%APS |
| --- | --- | --- | --- | --- | --- | --- | --- |
|  | Concentration (mM) | Volume (mL) | | | | | |
| Separating gel  (10%) | 4.0 | 0.003 | 2.05 | 1.65 | 1.25 | 0.05 | 0.05 |
| Stacking gel  （5%） | 6.7 | 0.004 | 2.7 | 0.67 | 0.5 | 0.04 | 0.04 |

* 30% acrylamide-bisacrylamide solution.

$ Separating gels were prepared with 1.5 M Tris-HCl (pH 8.8), and stacking gels were prepared with 1.0 M Tris-HCl (pH 6.8).

**Table S2. Formulations of SDS-PAGE gels containing different concentrations of TEEDA.**

|  | TEEDA | | ddH_2_O | 30% ACR^*^ | Tris-HCl ^$^ | 10%SDS | 10%APS |
| --- | --- | --- | --- | --- | --- | --- | --- |
|  | Concentration (mM) | Volume (mL) | | | | | |
| Separating gel  (10%) | 1.2 | 0.00129 | 2.05 | 1.65 | 1.25 | 0.05 | 0.05 |
|  | 2.4 | 0.00258 |  |  |  |  |  |
|  | 3.0 | 0.00322 |  |  |  |  |  |
|  | 3.6 | 0.00386 |  |  |  |  |  |
|  | 4.1 | 0.00445 |  |  |  |  |  |
|  | 4.8 | 0.00515 |  |  |  |  |  |
|  | 6.0 | 0.00643 |  |  |  |  |  |
|  | 7.1 | 0.00772 |  |  |  |  |  |
|  | 8.3 | 0.009 |  |  |  |  |  |
| Stacking gel  （5%） | 6.8 | 0.0059 | 2.7 | 0.67 | 0.5 | 0.04 | 0.04 |
|  | 7.5 | 0.0065 |  |  |  |  |  |
|  | 8.1 | 0.0070 |  |  |  |  |  |
|  | 10.4 | 0.009 |  |  |  |  |  |
|  | 11.6 | 0.010 |  |  |  |  |  |
|  | 17.4 | 0.015 |  |  |  |  |  |

* 30% acrylamide-bisacrylamide solution.

$ Separating gels were prepared with 1.5 M Tris-HCl (pH 8.8), and stacking gels were prepared with 1.0 M Tris-HCl (pH 6.8).

**Table S3. Formulations of SDS-PAGE gels containing different concentrations of SBS in the TEEDA-SBS-APS initiation system**

|  | SBS^*^ | | ddH_2_O | TEEDA | 30%ACR^$^ | Tris-HCl^^^ | 10%SDS | 10%APS |
| --- | --- | --- | --- | --- | --- | --- | --- | --- |
|  | Concentration (w/v) | Volume (mL) | | | | | | |
| Separating gel  (10%) | 0% | 0 | 2.05 | 0.0044 | 1.65 | 1.25 | 0.05 | 0.05 |
|  | 0.01% | 0.0025 | 2.0475 |  |  |  |  |  |
|  | 0.03% | 0.0075 | 2.0425 |  |  |  |  |  |
|  | 0.05% | 0.0125 | 2.0375 |  |  |  |  |  |
|  | 0.1% | 0.025 | 2.025 |  |  |  |  |  |
|  | 0.3% | 0.075 | 1.975 |  |  |  |  |  |
|  | 0.5% | 0.125 | 1.92 |  |  |  |  |  |
|  | 1% | 0.25 | 1.8 |  |  |  |  |  |
|  | 2% | 0.5 | 1.55 |  |  |  |  |  |
|  | 3% | 0.75 | 1.3 |  |  |  |  |  |
|  | 7% | 1.75 | 0.3 |  |  |  |  |  |
| Stacking gel  （5%） | 0% | 0 | 2.7 | 0.0059 | 0.67 | 0.5 | 0.04 | 0.04 |
|  | 0.01% | 0.002 | 2.698 |  |  |  |  |  |
|  | 0.03% | 0.006 | 2.694 |  |  |  |  |  |
|  | 0.05% | 0.01 | 2.69 |  |  |  |  |  |
|  | 0.1% | 0.02 | 2.68 |  |  |  |  |  |
|  | 0.3% | 0.06 | 2.61 |  |  |  |  |  |
|  | 0.5% | 0.1 | 2.6 |  |  |  |  |  |
|  | 1% | 0.2 | 2.5 |  |  |  |  |  |

* The indicated SBS volumes were added from a 20% (w/v) sodium bisulfite stock solution.

$ 30% acrylamide/bisacrylamide solution (29:1).

^ Separating gels were prepared with 1.5 M Tris-HCl (pH 8.8), and stacking gels were prepared with 1.0 M Tris-HCl (pH 6.8).
